# Strengthening health research capacity to address adolescent fertility in Northeast Brazil

**DOI:** 10.7189/jogh.09.010303

**Published:** 2019-06

**Authors:** Saionara MA Câmara, Tetine Sentell, Diego G Bassani, Marlos R Domingues, Catherine M Pirkle

**Affiliations:** 1 Faculty of Health Sciences of Trairi, Federal University of Rio Grande do Norte, Santa Cruz, Brazil; 2 Office of Public Health Studies, University of Hawaiʻi at Mānoa, Honolulu, Hawaiʻi, USA; 3 The Hospital for Sick Children, University of Toronto. Toronto, Canada; 4 University of Pelotas, Pelotas, Brazil

Locally-led health research is essential for overcoming local health challenges and for providing evidence for policy-maker decision-making, specific to where they live [1]. To adequately conduct such research requires sufficient local research capacity. However, despite greater health needs, usually lower-income settings have the least research capacity. Many barriers, such as underinvestment in universities and research centers and poor career prospects for researchers, prevent these places from achieving sufficient research potential to address their most pressing health problems [1]. Thus, research capacity strengthening is an essential goal for many low- and middle-income settings.

Brazil is an upper middle-income country, with the ninth largest economy in the world. However, across Brazil, health research capacity is unevenly distributed and concentrated in more economically developed areas. States such as Săo Paulo have research infrastructure and training programs comparable to North America, while other regions resemble poorer settings more reminiscent of the global south. According to 2016 data, the number of researchers with PhDs in the health sciences in the Northeast Region (6.7 researcher/100 000 people) was half that of the South or Southeast region (13.9 and 13.1, respectively) (http://estatico.cnpq.br/painelLattes/mapa/). In addition, while the overall number of researchers in the Northeast region of the country more than doubled between 2006 and 2016, the Brazilian government enacted severe cuts to research funding, threatening the recent gains [2].

Northeast Brazil—considered one of most iniquitous regions of this country – is characterized by high poverty, low educational attainment, and large gender inequalities. In 2018, the unemployment rate for those aged 18-24 living in Northeast Brazil was the highest of all the Brazilian regions (32.8%) [3]. In the state of Rio Grande do Norte, which is one of nine states in the Northeast and the focus of this team’s activities, approximately 25% of the population is categorized as poor and 10% as extremely poor [4]. As of 2010, less than 50% of 18-year-olds had completed primary school [4].

The issue of unevenly distributed health research capacity in Brazil becomes more evident when addressing the reproductive health needs of adolescents in the Northeast. Across Brazil, approximately 11% of girls aged 15 to 19 years have been pregnant [5]. Since the 1980s, fertility rates in Brazil have *dropped* among those 20 and older, but *increased* in those between 15 and 19 years [6]. Among very young adolescents, ages 10 to 14 years, fertility rates *decreased* from 2000 to 2012 for the more affluent regions of Brazil, but *increased* in North and Northeast regions [7]. There is limited research available to explain these trends, especially for very young adolescents. This situation exemplifies the inverse care law; the availability of health research capacity varies inversely with the need of the population served [8].

Research on adolescent fertility – that is, births to women 19 years and younger – has relevance to policy-making and economic development. Adolescent fertility intersects with poverty and gender norms. As observed elsewhere, adolescent childbearing in Brazil concentrates in the most disadvantaged populations [9]. According to the GRAVAD survey of adolescents from Porto Alegre, Rio de Janeiro, and Salvador, adolescent childbearing was common among those from low-income families, primary school dropouts, and those of black or mixed race [9]. Adolescent fertility also contributes to the perpetuation of poverty across successive generations of women. In one longitudinal study of Brazilian youth, women born to adolescent mothers were twice as likely to be adolescent mothers themselves [9]. Reducing adolescent fertility in Northeast Brazil may help break entrenched cycles of poverty, but to do so requires a solid understanding of the social, economic, and psychological motivations favoring early motherhood. In the absence of sufficient health research capacity, obtaining an adequate evidence base to effectively address adolescent fertility in Brazil is challenging.

## RESEARCH INITIATIVE TO BUILD CAPACITY

Research capacity strengthening is essential for economically disadvantaged regions of Brazil, such as the Northeast. Research capacity strengthening is defined as any efforts to increase the ability of individuals and institutions to undertake high-quality research and to engage with the wider community of stakeholders [1], including the national and international research community, health care providers, and policy-makers. Efforts may encompass professional development programs, organizational initiatives with research groups or institutions, and activities to support research governance and structures [1].

In the fall of 2016, the National Institutes of Health (NIH) awarded the authors an exploratory and developmental research grant from the John E. Fogarty International Center. This funding supports locally-relevant and catalytic research on non-communicable diseases, through projects that contribute to the ‘long term goals of building sustainable research capacity’. Capitalizing on existing relationships in Brazil, Canada, and the United States, and a previous history of collaboration investigating links between adolescent childbearing and chronic conditions, the authors successfully developed a competitive project that dually supports capacity-building and addresses a research gap of importance to Brazil. The activities focus on professional development and supporting research at a newly formed university in rural Northeast Brazil.

**Figure 1:**
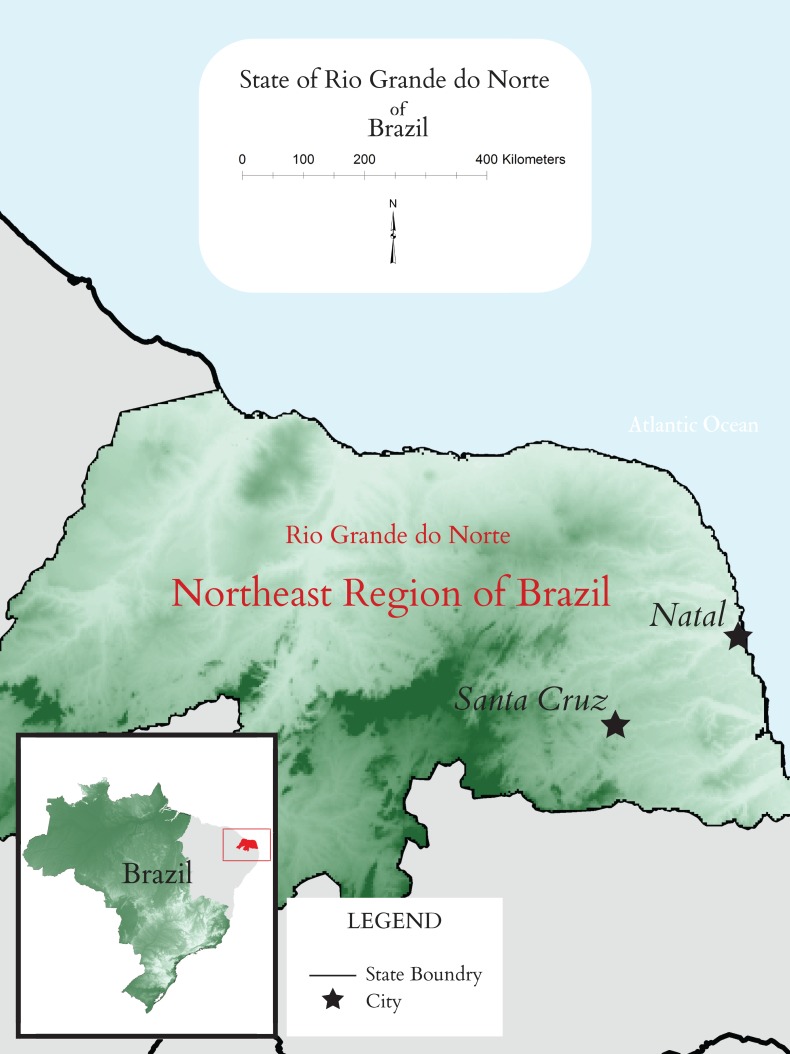
Map of the Rio Grande do Norte State, Northeast Brazil.

The project’s goal of capacity building fits well with the objectives of the Brazilian partner university. The Federal University of Rio Grande do Norte (UFRN) established a satellite campus in Santa Cruz in 2008, called FACISA, which is approximately 100 km from the urban, principal campus in the state’s capital, Natal. With approximately 40 000 inhabitants, Santa Cruz is the main city of the rural Trairi region of Rio Grande do Norte (see **Figure 1**). The geography of Trairi presents significant obstacles to students seeking higher education, as it is sparsely populated and covers large geographic distances. Yet, lessons learned from Brazil on how to deliver quality higher education to geographically isolated areas could benefit other rural and remote global communities facing similar constraints. Given limited economic opportunities, this relatively new campus in Santa Cruz, with 700 students, specifically aims to stimulate economic growth and strengthen health infrastructure by providing training for well-paid health sciences’ careers.

### Capacity strengthening activities

Following the award of funding, the authors, supported by a strong research team and institutional environment, launched a series of capacity-strengthening activities, which are described below, for this rural campus in Northeast Brazil. Their aim is to use this capacity to develop a comprehensive research platform focused on the health of young women in rural Brazil. This includes improving the knowledge base of local drivers of adolescent pregnancy, the immediate and long-term health risks of adolescent pregnancy and childbirth, and programs that can support adolescents in the region.

#### Objective 1: Train students in best practices for the conduct of epidemiological research

The authors developed 13 specialized training modules of three to six hours each that were taught as night courses by local and international academics over three months in spring of 2017. Eight courses emphasized skills needed for field epidemiological data collection such as survey administration and taking anthropometric and clinical measures. Five more modules provided in-depth training on ethics in research, focusing on vulnerable populations (children and pregnant women). The materials taught were informed by the authors’ experiences hiring research staff and designed to complement content traditionally taught in health sciences degrees with practical skills for carrying out quantitative health research.

Thirty mostly undergraduate FACISA students took the optional course. Twenty-three students completed the training modules. Students who completed 75% the modules were eligible to apply to be interviewers on a small prospective epidemiological study of young pregnant women. Twelve students applied for the interviewer positions; four were hired as interviewers and one as a recruiter. As an indicator of success, the trainees were able to successfully interview 100 pregnant women from rural Trairi with retention of >90% (see objective 3).

Seventeen students completed a satisfaction questionnaire at the end of the course and all indicated appreciation of the course and its structure; learned new material from it; and believe the course would help in their future professional endeavors. Nearly all students affirmed that the information from the course helped them in their other classes and the training modules encouraged them to think about different opportunities in public health. Students also expressed appreciation of the international guest speakers and their novel teaching methodologies. The only negative aspect raised by the students was that the modules clashed with some of their other academic obligations. Lessons learned from this experience, especially with regard to sustainability, include a high need to formalize the course as part of the FACISA curriculum, as well as a need for technical solutions that would allow international guest lecturers to participate from a distance. Aligning the training modules with practical applied research skills was a critical success of the program, evidenced by over two-dozen students participating, despite receiving no academic credit.

#### Objective 2: Provide graduate students with international training opportunities

The authors leveraged the Fogarty Grant to obtain additional funds from Brazilian resources to support international training opportunities. These funds supported travel of 4 graduate students to the University of Hawaiʻi at Mānoa to work on manuscripts with US-based researchers. Another graduate student attended a networking meeting at the NIH in Bethesda, Maryland. Additionally, 1 MSc student visited the Center for Epidemiological Research in Pelotas, in Southern Brazil.

Cumulatively, the students worked on five manuscripts during their visits, and all are under-review in high quality international journals. All the graduate students completed their degrees. Three graduate students were offered academic appointments, another is working as a research assistant, and the fifth was hired by the state Secretary of Public Health.

**Figure:**
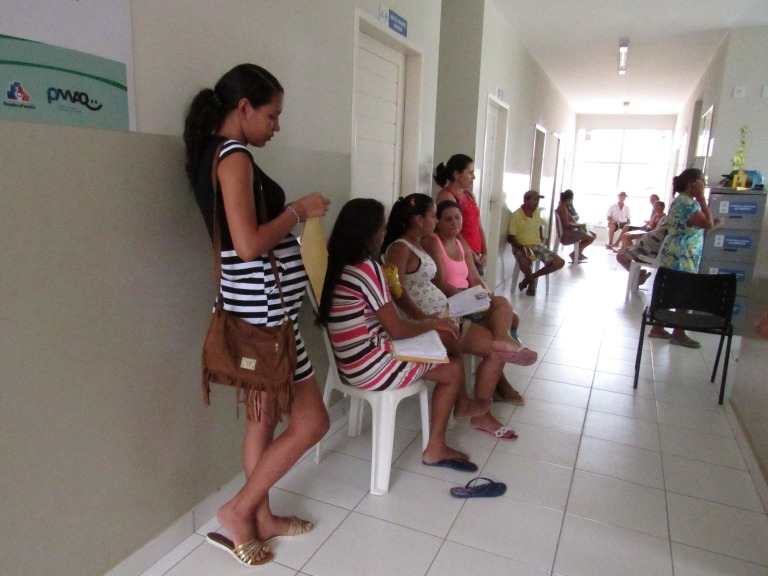
Photo: A project participant, used with consent

Overall, the foreign training opportunities and co-authorship with international academics improved the students’ professional competitiveness and support long-term health research capacity in Brazil. Given that over 85% of PhD graduates in Northeast Brazil remain in the region, which is the highest retention rate in the country, highly trained graduates contribute to long-term health research advancements [10].

#### Objective 3: Employ students trained by the project in a prospective, longitudinal pilot study of pregnant young women in the Trairi region

Students were given the opportunity to apply the research skills through hands-on employment in a longitudinal study. Under the supervision of the local principal investigator, the students were able to participate in the full cycle of data collection on a challenging research population and topic. Those hired by the project recruited and consented pregnant women for a study documenting drivers and consequences of early pregnancy. The interviewers conducted two pregnancy and one post-partum interviews, took anthropometric and clinical measures, blood and urine samples, and conducted tests of physical function. The authors conducted regular quality control assessments and evaluated the professionalism of the interviewers. No issues have been identified.

## FINAL CONSIDERATIONS AND FUTURE PLANS

By supporting health research capacity in Northeast Brazil, this project builds knowledge about adolescent fertility and lays the foundation for future research in an underserved region. Our activities will contribute to ensuring a qualified workforce for local epidemiological research; providing skills to students from FACISA to competitively apply for research positions there and elsewhere; supporting the academic development of students interested in graduate school and academic careers; providing international exposure to students from a rural region and; increasing health research proficiency and capacity.

Finally, this work is occurring in an era characterized by threats to prevention-focused public health programs. Following recent political changes in Brazil, slashes to funds for current and future social programs are expected. There is also a growing political movement in Brazil to reduce access to family planning and instate ultra-conservative social policies, which in many ways mirror the political environment of the US Recent expansion of the Reagan-era Mexico City Policy, or Global Gag Rule, raises considerable uncertainties about who and what programs will be targeted by the policy and may result in a fear of collaborating with US partners, especially in reproductive health. Without sufficient research capacity, and as existing capacity is threatened by national and global policy shifts, important recent global health gains may be jeopardized.
